# Supplementary material for: Conditional chemoconnectomics (cCCTomics) as a strategy for efficient and conditional targeting of chemical transmission
Source: eLife. 2024 Apr 30;12:RP91927. doi: 10.7554/eLife.91927 (PMC11060718; doi:10.7554/eLife.91927)
Supplement: Figure 3—source data 3. [file elife-91927-fig3-data3.zip › Figure 3 source data3 part 2.zip!/Figure 3-Source data 3-part2/1052-13-CRISPResso_Report_ejedac2.zip!/CRISPResso_on_1052_16/3a.Indel_size_distribution.pdf]

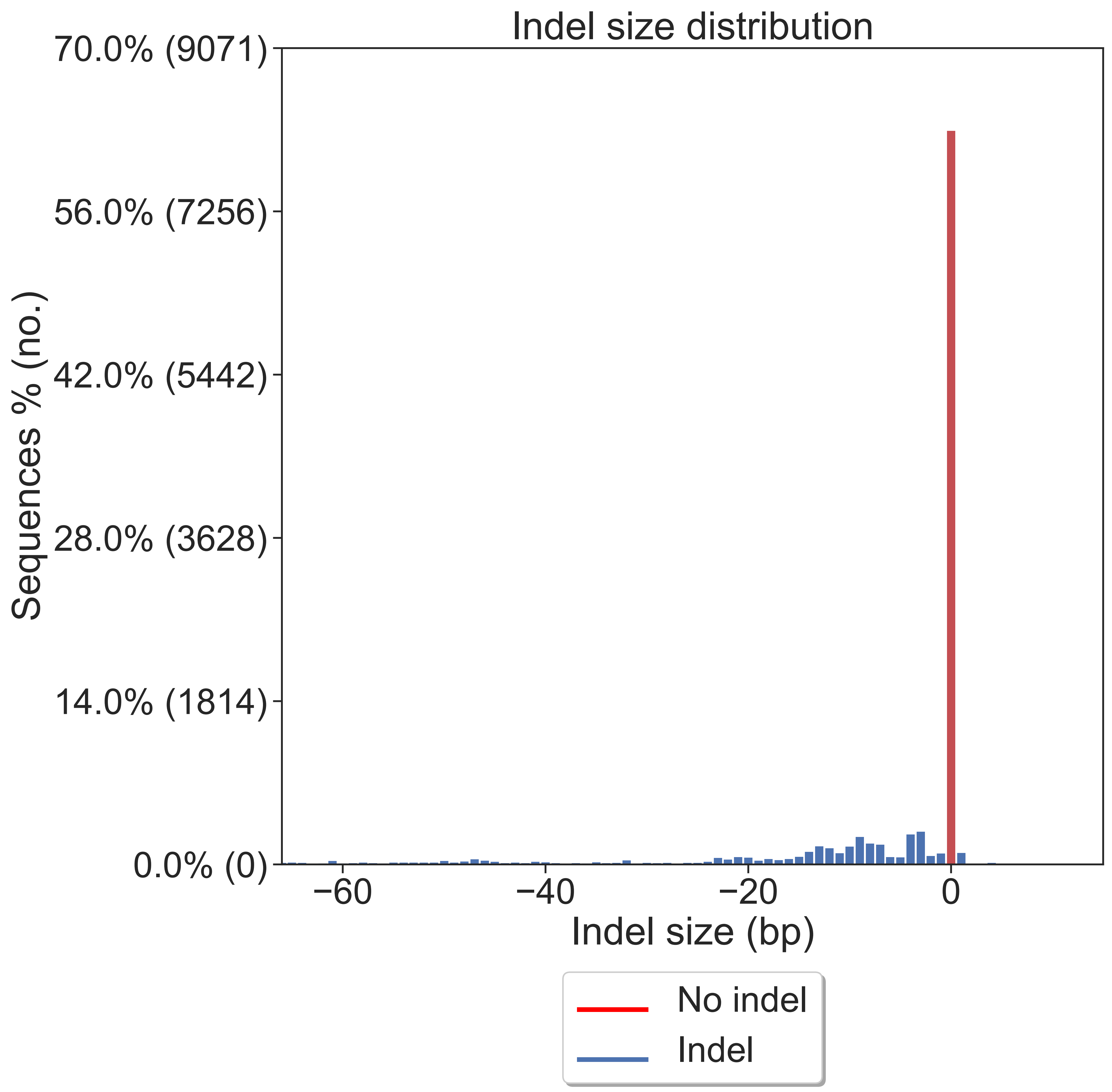

Indel size distribution
70.0% (9071)
56.0% (7256)
42.0% (5442)
28.0% (3628)
14.0% (1814)
0.0% (0)
Sequences % (no.)
−60
−40
−20
0
Indel size (bp)
No indel
Indel
